# A community outbreak of Legionnaires’ disease caused by outdoor hot tubs for private use in a hotel

**DOI:** 10.3389/fmicb.2023.1137470

**Published:** 2023-04-25

**Authors:** Mercedes Gumá, Vladimir Drasar, Beatriz Santandreu, Rosa Cano, Baharak Afshar, Antonio Nicolau, Magdalena Bennassar, Jorge del Barrio, Pau Crespi, Sebastian Crespi

**Affiliations:** ^1^Conselleria de Salut i Consum, Govern Balear, Palma de Mallorca, Spain; ^2^Public Health Institute Ostrava, National Legionella Reference Laboratory, Ostrava, Czechia; ^3^Environmental Health and Laboratory Services, Biolinea Int., Palma de Mallorca, Spain; ^4^Centro Nacional de Epidemiología and CIBERESP, Instituto de Salud Carlos III, Madrid, Spain; ^5^Respiratory and Vaccine Preventable Bacteria Reference Unit (RVPBRU), UK Health Security Agency (UKHSA), London, United Kingdom

**Keywords:** Legionnaires’ disease, community outbreak, outdoor hot tubs, hotel, outbreak investigation

## Abstract

During the period October–November 2017, an outbreak of Legionnaires’ disease involving 27 cases occurred in the tourist area of Palmanova (Mallorca, Spain). The majority of cases were reported by the European Centre of Disease Prevention and Control (ECDC) as travel associated cases of Legionnaires’ disease (TALD). Most cases belonged to different hotel cluster alerts. No cases were reported among the local population residing in the area. All tourist establishments associated with one or more TALD cases were inspected and sampled by public health inspectors. All relevant sources of aerosol emission detected were investigated and sampled. The absence of active cooling towers in the affected area was verified, by documents and on-site. Samples from hot tubs for private use located on the terraces of the penthouse rooms of a hotel in the area were included in the study. Extremely high concentrations (> 10^6^ CFU/l) of *Legionella pneumophila*, including the outbreak strain, were found in the hot tubs of vacant rooms of this hotel thus identifying the probable source of infection. Meteorological situation may have contributed to the geographical distribution pattern of this outbreak. In conclusion, hot tubs for private use located outdoors should be considered when investigating community outbreaks of Legionnaires’ disease of unclear origin.

## 1. Introduction

Legionnaires’ disease (LD) is a respiratory illness caused mainly by inhalation of aerosolized water contaminated by *Legionella* bacteria. These bacteria are found in both natural and artificial water systems. In addition, *Legionella* can also cause Pontiac fever, an acute febrile, self-limiting, and flu-like illness. Outbreaks of LD have been most frequently linked to cooling towers or evaporative condensWPers, hot water systems and spa-type pool systems although other sources, like humidifiers, ornamental fountains, cooling misting devises, irrigation systems, and others have been identified (World Health Organization, 2007; European Center for Disease Prevention and Control, 2022).

Cases of LD are categorized as being community, travel or hospital acquired based on the type of exposure. For travel-associated cases, experience has shown that clusters initially associated with a certain hotel are actually cases of a more far-reaching community outbreak. Inversely, community-acquired outbreaks can involve international travellers living in other countries. In both cases, exchanging information between the local, national health authorities of the affected countries and ECDC may be of great interest for identifying the origin of the outbreak (Decludt et al., 1999).

Bathing and recreational waters, in their various types, have often been implicated in outbreaks of LD and Pontiac fever. Hot tubs, also called spa pools, whirlpools and whirlpool spas, have been linked to *Legionella* infections, especially when poorly maintained (World Health Organization, 2006). The outbreaks have almost always occurred indoors affecting users or people in the same building area or in close proximity. In these cases, outbreaks can be very large, such as the one that occurred in 1999 in the Netherlands, which affected many visitors to a flower show (Den Boer et al., 2002). When they have occurred outdoors, they have affected a smaller number of people, mostly the users themselves or people that were very close to the source pool (Leoni et al., 2018; Hlavsa et al., 2021).

We describe here a widely dispersed LD community outbreak that almost exclusively affected international visitors to various hotels in a tourist area of Mallorca, Spain, caused by hot tubs for private use located on the terraces of the penthouses of a hotel in the area. The outbreak followed a geographical distribution pattern somewhat reminiscent of those described in different outbreaks caused by cooling towers. The outbreak was investigated to determine the source of infection, to stop further transmission and to prevent further cases. To our knowledge, this is the first community outbreak caused by outdoor hot tubs that has affected a wide geographical area and highlights the importance of considering these systems when investigating community outbreaks of uncertain origin.

## 2. Methods

### 2.1. Setting and summary history of the outbreak

Palmanova, a small town, located by the Mediterranean Sea, is one of the most popular tourist destinations on the island of Mallorca. It has a wide variety of tourist accommodation (hotels, aparthotels, and tourist apartments) and leisure areas, all in an area of approximately 1.5 Km^2^.

Between 4th October 2017 to 16th November 2017, twenty-seven cases of LD associated with the area of Palmanova were reported to the Spanish Health Authorities. Most cases were reported by ECDC as TALD in different cluster alerts. The ECDC published a rapid risk assessment report about the outbreak on the 23rd October 2017 (European Center for Disease Prevention and Control, 2017).

### 2.2. Epidemiological investigation

All cases except three were notified to the Spanish Health Authorities by the European Legionnaires’ Disease Surveillance Network (ELDSNet) according to standardized procedures (European Center for Disease Prevention and Control, 2012). ELDSNet is an ECDC surveillance scheme. The data reported included, as a minimum, date of onset of symptoms, dates of stay and detail of the accommodation site. In addition, the health authorities of some member states sent directly to the Spanish National Epidemiology Center, additional information on cases, useful for the investigation of the outbreak, e.g., places visited during their stay. Three cases were reported by local Spanish hospitals to the National Epidemiological Surveillance Network following national regulations (Ministerio de Sanidad, Servicios Sociales e Igualdad, 2015). Confirmed and probable case definitions in accordance with the ECDC were used (Official Journal of the European Union, 2008) with an onset date after 1 September 2017 and a history of staying in or visiting the Palmanova area in the 2–10 days before onset of disease. No further epidemiological investigations were carried out at national level.

### 2.3. Environmental investigation

All tourist establishments associated with one or more TALD cases were inspected and sampled by public health inspectors. The locations of the accommodation sites were georeferenced using visual pinpointing in Google Earth and distances were calculated with the same tool. Possible incidents in the operation of the municipal water distribution network, which was also sampled, were investigated. All relevant sources of aerosol emission detected were investigated and sampled: public and private sprinkler irrigation systems, ornamental fountains, beach showers, street cleaning vehicles, and a car wash station. An active search was carried out, with the help of the local police, of other possible sources of aerosol emission, including misting cooling units that had been in operation during the outbreak period and the previous month. The absence of active cooling towers in the affected area was verified by documents and on-site. Finally, samples of hot tubs for private use located on the terraces of the penthouse rooms of a hotel in the area, Hotel A, were included in the study.

Weather data corresponding to the month of September 2017, were retrospectively retrieved from two meteorological stations, Calvià and Son Ferriol, through the tool SIAR (Spanish Agroclimatic Information System for Irrigation).^1^ The data on winds were retrieved through specialized portals from the weather station of a nautical club in the area of Palmanova and from the Port of Palma de Mallorca.^2^

### 2.4. Microbiological investigation

#### 2.4.1. Clinical specimens

Urine and lower respiratory samples (if available) from cases travelled from United Kingdom were sent to the Respiratory and Vaccine Preventable Bacteria Reference Unit (RVPBRU), UKHSA, for confirmation, detection and typing. Lower respiratory tract samples were only available for six cases. These samples were tested by a *Legionella pneumophila* serogroup Sg 1 qPCR assay (Mentasti et al., 2015). Sequence Based Typing (SBT) was performed on *L. pneumophila* Sg 1 isolates obtained from two cases (Gaia et al., 2005; Ratzow et al., 2007). Nested SBT was performed on direct clinical samples from the remaining four cases in the absence of isolates (Fry et al., 2006). SBT data were analyzed using the web-based *L. pneumophila* SBT database.^3^ This link is undergoing development and is currently unavailable externally but can be accessed internally by the database curators at UKHSA.^4^

Information regarding clinical samples from other cases (non-UK cases) was received from ELDSNet or provided by the local hospitals that reported the cases. SBT data from one clinical *L.pneumophila* Sg1 isolate was received from the National Legionella Reference Laboratory (Spain).

#### 2.4.2. Environmental samples

Environmental samples were collected following standardized protocols (Ministerio de Sanidad y Consumo, 2003) and were analyzed for *Legionella* spp. according to UNE-ISO 11731:2007 – *Water quality-Detection and enumeration of Legionella standard*. The isolates were further identified and serotyped (*L. pneumophila* Sg 1, *L. pneumophila* Sg 2–14 and *Legionella* non-pneumophila species) using latex agglutination reagents (Oxoid, Spain). Further monoclonal antibody (MAb) typing was carried out on the *L. pneumophila* Sg 1 isolates, using an international panel of seven monoclonal antibodies (Dresden panel) (Helbig et al., 1997, 2002).

Genotyping on selected strains (two from each MAb type identified) was performed according to the seven gene protocol from ESCMID Study Group for Legionella Infections (ESGLI) consensus Sequence-Based Typing (SBT) scheme (Gaia et al., 2005; Ratzow et al., 2007) at the National Legionella Reference Laboratory (Czech Republic). The online *L. pneumophila* SBT sequence quality tool was used to determine individual sequence type (ST) for each sample. The standardized protocols including the amplification and sequencing primers for *L. pneumophila* SBT and direct nested SBT, are available at the website https://bioinformatics.phe.org.uk/legionella/legionella_sbt/php/sbt_homepage.php. This website is undergoing development and is currently unavailable externally but can be accessed internally by the database curators at UKHSA (see text footnote 4).

### 2.5. Control measures

Control measures started on the 6th October 2017, after the outbreak was declared. The following actions were taken in the neighbourhood: the public and private irrigation systems using sprinklers were shut down; the use of street cleaning vehicles was stopped; an urban fountain, a car washing station and the beach showers were closed; one hotel was closed down after deficiencies were detected and several water samples taken at the premises were positive for *Legionella* spp. Hot tubs on the terraces of the vacant rooms were drained and taken out of service. All premises involved in the investigation were ordered to clean and disinfect the closed systems and re-test before reopening, according to local regulations (Ministerio de Sanidad y Consumo, 2003).

## 3. Results

### 3.1. Epidemiology

**Figure 1:**
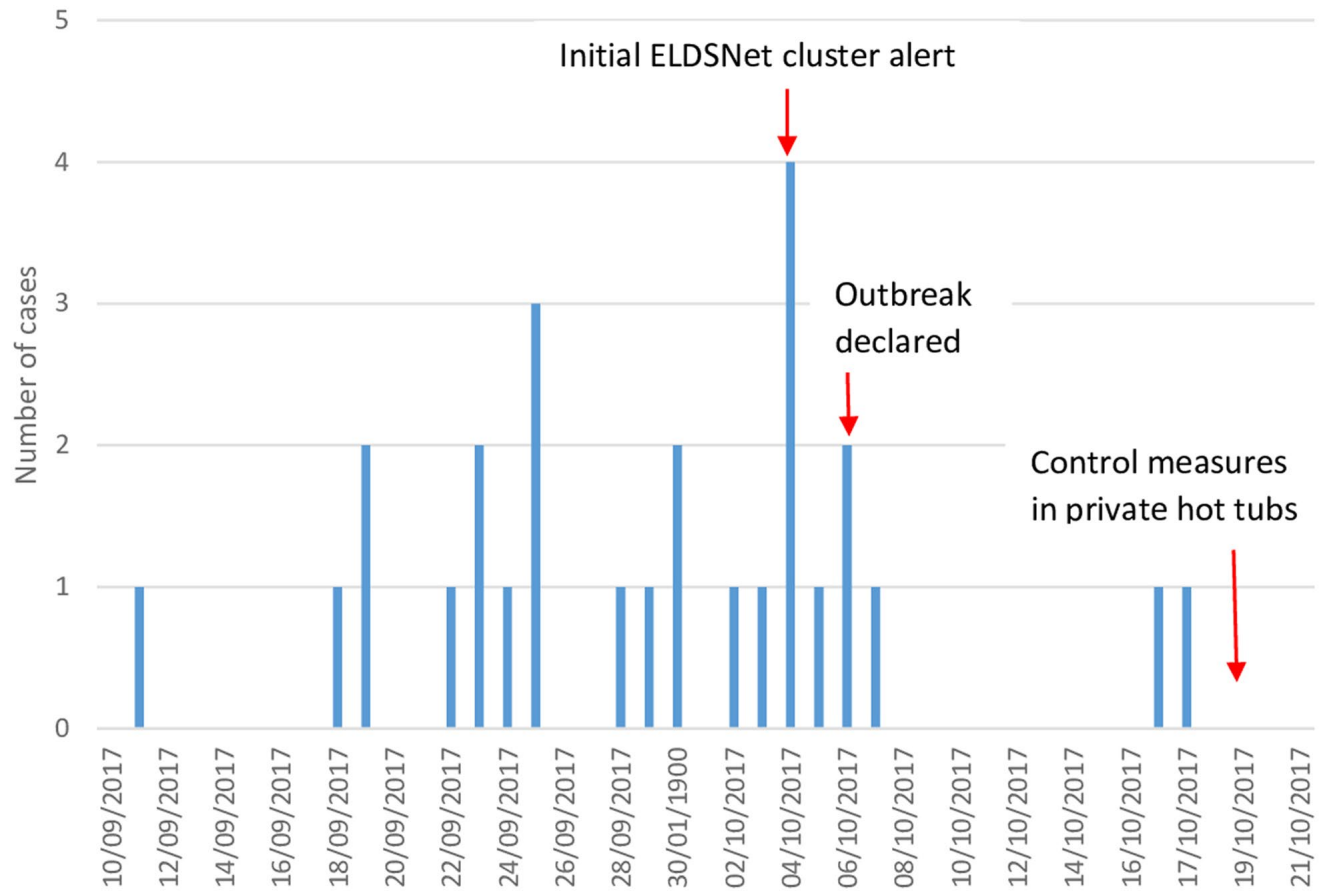
Distribution of cases of Legionnaires’ disease by date of symptom onset (*n* = 27).

Twenty seven cases of Legionnaires’ disease, including a fatal case, were reported during this outbreak (26 confirmed cases and 1 probable case). Twenty four cases were diagnosed outside Spain, upon return of the cases to their country of origin. Cases were reported by the United Kingdom (18), Denmark (2), France (2), Czech Republic (1) and Sweden (1). The cases were aged between 46 and 87 years and include 15 males and 12 females. The illness onset occurred between 11th September 2017 and 17th October 2017. All cases except one were tourists staying in ten different hotels or tourist apartments in the affected area. One case was working at a hotel not previously related to this cluster (Figure 1).

**Figure 2:**
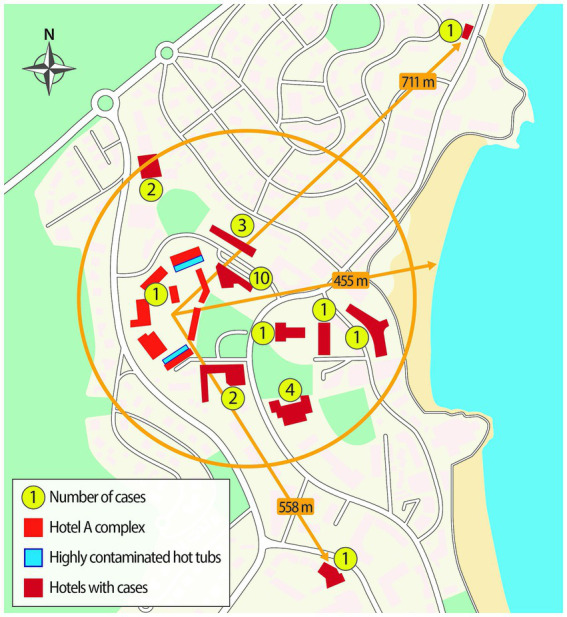
Schematic diagram of the affected area, accommodation sites associated with cases, and some selected distances.

One of the hotels was associated with 10 cases, one with four, one with three, two with two cases, and the rest with one case. All hotels associated with cases were located within a radius of about 500 meters from what was considered initially the epicenter of the outbreak, i.e., the hotel associated with 10 cases (Figure 2).

### 3.2. Environmental findings

Inspections of potential communal sources (public showers on the beach, ornamental fountains, sprinkler irrigation systems, car wash stations) did not reveal any deficiencies. One of the hotels presented some structural and operational deficiencies in the drinking water system (high corrosion levels in hot water tanks, low hot water temperatures).

Hotel A was located next to the hotel associated with 10 cases and consisted of seven buildings with three or four floors. Each building had penthouses on the top floor with hot tubs on the terraces. These hot tubs are for the exclusive use of the guests staying in these rooms. At the time of the visit, on the 18 and 19 October 2017, there were seventy five hot tubs with a volume of 1.1 m^3^ water each, but only fifty nine were in use. The hot tubs were equipped with a recirculation pump, a heater and multiple water jets with passive air mixing. The water was treated manually with bromine tablets put into the filter housing. The average water temperature was 32.7°C. The average total bromine level was 2.1 ppm (required range by Spanish regulations: 2.0–5.0 mg/L).

At the time of the visit, the occupancy rate for the penthouse rooms was about 50%. In the vacant rooms, the hot tubs were left filled with water but without regular checks or maintenance. They were connected to the main electrical switch in the room thus implying that there was no recirculation or treatment when not in use. We found 59% of them with bromine levels <2.0 ppm.

In September 2017, the average temperature in Son Ferriol was 21.3°C (range: 13.2°C–30.7°C). The relative humidity fluctuated between 21.4 and 98.6% with an average of 73.1% and rainfall was <1 mm. In Port of Palma, the wind speed was generally low, less than 6.1 m/s, and the direction of the wind was variable, prevailing N, S, SW, and NE. In the Calvià station, the average temperature in the last 10 days of September was 20.6°C and the average relative humidity was 79.4%. At the Palmanova nautical club station, the prevailing annual winds were S, SW, NE, and NW, being generally light, with a speed of less than 6.1 m/s.

### 3.3. Microbiological results

Twenty-six cases were laboratory confirmed by urinary antigen and one case by qPCR detection. One clinical isolate (Case 12; Table 1) was identified as *L. pneumophila* Sg 1, MAb type France/Allentown, ST82. *L. pneumophila* serogroup 1, ST 82 isolates were also obtained from two cases travelled from United Kingdom (Case 8 and Case 11; Table 1). Although *L. pneumophila* was not isolated from four additional cases, nested SBT on direct clinical samples yielded partial allelic profile consistent with ST 82 Table 1.

**Table 1:** SBT profiles and MAb types of the relevant clinical specimens and environmental strains.

Clinical specimens				
Origin	SBT (allelic profile)	Isolate	DNA extract	MAb Type
Case 2^a^	SBT: undetermined (5,0,0,10,6,10,6)		Yes	
Case 8^a^	ST 82 (5,1,22,10,6,10,6)	Yes		NT^b^
Case 9^a^	SBT: undetermined (5,0,0,10,6,10,6)		Yes	
Case 11^a^	ST 82 (5,1,22,10,6,10,6)	Yes		NT^b^
Case 12^c^	ST 82 (5,1,22,10,6,10,6)	Yes		France/Allentown
Case 16^a^	SBT: undetermined (5,0,0,10,6,10,6)		Yes	
Case 18^a^	SBT: undetermined (0,0,0,0,0,0,6)		Yes	
**Environmental strains**				
	**SBT (allelic profile)**	**Isolate**		**MAb Type**
Hot Tub^d^ (Hotel A)	ST 82 (5,1,22,10,6,10,6)	Yes		France/Allentown

a Sample tested at RVPBRU, UKHSA.

b Not tested.

c Sample tested at National Legionella Reference Laboratory, Spain.

d Sample tested at National Legionella Reference Laboratory, Czech Republic.

146 water samples from different potential sources initially suspected were collected and tested for *Legionella* spp. (122 samples from domestic water systems of the hotels associated with cases, seven from street cleaning vehicles, six from irrigation water systems, six from misting cooling units, four from aquatic parks, ornamental fountains, and beach showers and one from a car washing station). Nineteen samples yielded positive results to *L. pneumophila* sg1 (range: < 400 CFU/l - > 10^6^ CFU/l). These isolates were identified as MAb type OLDA, OLDA/Bellingham, or Benidorm that were distinct from the LD cases (Mab type France/Allentown) Table 1.

Additionally, 48 water samples were taken from the hot tubs at the penthouses of the hotel A. Positive results to *L. pneumophila* Sg 1 were obtained in 38 samples. Twelve samples were from hot tubs in vacant rooms, the concentration of *Legionella* spp. was >10^6^ CFU/l. In the rest of the samples, *Legionella* spp. concentrations of 10^2^–10^3^ CFU/l were obtained (parametric value in Spanish regulations: <10^2^ CFU/l). *L. pneumophila* serogroup 1, MAb (France/Allentown), ST82 was identified mixed with OLDA/Bellingham strains Table 1.

No positive results were obtained from samples collected from any of the other sampled systems (drinking water, irrigation, outdoor recreational pool, and indoor hydrotherapy pool) of Hotel A.

## 4. Discussion

The hot tubs for private use on the upper terraces of penthouse rooms of Hotel A were the most likely source of this community outbreak. Both, the environmental findings (a plausible source, located just beside the epicenter of the outbreak) and the microbiological findings (high proportion of positive systems, extremely high concentrations of *Legionella* in significant proportion of the hot tubs and identical MAb and sequence type as the outbreak strain) strongly support this conclusion.

This outbreak is extraordinary in different aspects. First, to our knowledge, this could be the first outbreak of LD caused by outdoor hot tubs affecting a wide geographical area. *Legionella* infections in non-users (visitors to the premises where the hot tubs are located, or people exposed nearby) have been well documented in different outbreaks both indoors and outdoors but outbreaks covering a large distance have not been previously reported. This outbreak involved cases staying in hotels located in a radius of about 400 meters from the source, with two exceptions located at 558 m and 711 m, respectively. Almost all the affected hotels were distributed in a circular sector whose radii are oriented to the NE and SE of Hotel A, respectively. There are no cases to the west, as the hotel is located on the western limit of the urbanized area. Not surprisingly, about half of the cases occurred in hotels located adjacent to Hotel A, one with 10 cases, a second with three cases and a third with two cases. However, one of the affected hotels, located just over 300 m from the source hotel, had four affected and another located about 200 m away had 2 cases. It is unlikely that these last six cases would have approached the Hotel A, that was on the outskirts of town and away from the beach. In this sense, the pattern of distribution of cases of this outbreak is very reminiscent of patterns seen in outbreaks caused by cooling towers where bioaerosols can travel hundreds of meters from the source (Sabria et al., 2006; Ferré et al., 2009; Weiss et al., 2017; Dyke et al., 2019) rather than outbreaks caused by hot tubs where typically only users or people staying in their vicinity are affected (Benkel et al., 2000; Götz et al., 2001; Den Boer et al., 2002; Coetzee et al., 2012). This could be due to several reasons. In some hot tubs, the water level dropped below the nozzles and the water jets were directed into the air, causing greater aerosolization. We speculate that this, together with the high number of contaminated hot tubs and the high concentration of *Legionella* in the water combined with the location on the rooftop terraces and dispersal by wind, may explain the dissemination pattern of the aerosols. The case location data are consistent with the pattern of atmospheric distribution that would be expected in such a case: more cases in the vicinity and fewer as we move further from the source.

Second, the high proportion of hot tubs colonized and the very high concentrations of *Legionella* spp. in many of them is surprising. An extensive study of the prevalence of *Legionella* in various water systems of hotels in the Balearic Islands has found that 10.9% of the hot tubs were contaminated with *Legionella* (Doménech-Sànchez et al., 2022). Another study in Quebec found that *Legionella* spp. was detected in 23% of public whirlpool spas (Brousseau et al., 2013). These data are still very far from the 79.1% colonized systems that we found in the Hotel A. The hot tubs were contaminated with the same strain, that was not found anywhere else. A plausible explanation for contamination is by bioaerosols emitted by the contaminated hot tubs seeding adjacent or nearby terraces. The terraces of the penthouses were separated by 1.5 m high walls, although this was reduced to approximately 1 m at the outer edge. As the hot tubs were 0.75 m in height and the distance between each hot tub was only a few meters, it is not difficult to imagine that the emitted aerosols could easily contaminate neighbouring hot tubs. In addition, contaminated hot tubs with high levels of *Legionella* spp. were almost all clustered in two of the seven blocks, suggesting again aerosol contamination. Contamination by other means, for example, through the maintenance staff or their equipment, is less likely since the hot tubs were not regularly maintained in the unoccupied penthouses. In addition, it is likely that stagnation and the reduced maintenance of the hot tubs in the vacant rooms contributed to the growth of the bacteria. In fact, bromine concentrations below required levels and mid-ranged water temperatures could have facilitated microbial growth. It is known that low halogen levels and poor maintenance increase the relative risk of *Legionella* colonization in spa-type pools (Brousseau et al., 2013; Papadakis et al., 2018).

According to the retrieved meteorological data, in September 2017, weather conditions could have favoured transmission and the duration of the outbreak. The average relative humidity was high. It has been reported that increased high humidity is positively associated with increased incidence of LD (Pampaka et al., 2022). Similarly, the winds speeds were low, that could lead to slower dispersion of aerosols. The variable wind direction and the prevailing winds, especially S and SW, are consistent with the pattern of geographic distribution of the cases. Different studies have suggested that climatic conditions are related to the risk of Legionella infection (Simmering et al., 2017; Walker, 2018) and meteorological studies have usually been included in investigations of legionellosis outbreaks caused by cooling towers or similar equipment (Kirrage et al., 2007; Nygård et al., 2008; Shivaji et al., 2014). To the best of our knowledge, this would be the first time that the analysis of weather conditions has been applied to an outbreak caused by hot tubs, albeit retrospectively.

Another notable aspect of this outbreak was that the hot tubs involved were for the private use of customers in penthouse rooms. This is important since in Spain, swimming pools and similar bathing systems for private use, even if they are in commercial buildings such as hotels and similar, were expressly excluded from the current regulations (Ministerio de Sanidad, Servicios Sociales e Igualdad, 2013), except for the notification of incidents and that their existence, number, and location must be notified before putting them into operation in the regional registry. In this way, these systems for private use were not necessarily subject to the same regulatory obligations as swimming pools and hot tubs for public or collective use, including regular maintenance and sampling. Interestingly, in recent years, the number of hotels that incorporate hot tubs for private use on room terraces has increased in general and the use of these systems has become popular both in the public and private settings. Extensive use of spa pools, combined with inadequate maintenance, has contributed to outbreaks of Legionnaires’ disease, which in many cases have originated in a hotel (Centers for Disease Control and Prevention, 2004; Hlavsa et al., 2021). Therefore, the implementation of guidelines and recommendations for good practices for the use and maintenance of hot tubs for private use could help prevent *Legionella* infections. Following this outbreak, the Health Authorities of the Balearic Islands published a guide for the hygienic maintenance of hydro massage vessels that also included those for private use (Govern Balear (GOIB), 2018).

No cases were reported among the local population residing in the area and most cases were British tourists, but this was a very popular with British tourist area (European Center for Disease Prevention and Control, 2017). The people who work in the area are mostly staff from hotels and commercial establishments, so work indoors and unlikely to be exposed. Local doctors are familiar with LD and local hospitals have access to urinary antigen testing. Therefore, it is unlikely that they missed cases among the native population, especially considering that the outbreak was a high profile and reported by the local media. In summary, the pattern of the affected population reflects the pattern of the exposed population well.

This outbreak also highlights the importance of the European surveillance scheme for cases associated with travel ELDSNet. The rapid notification of several clusters associated with hotels in the Palmanova area allowed the early identification of this community outbreak and the subsequent launch of an investigation and implementation of control measures. The exchange of relevant information by ELDSNet relating to the molecular profile of the outbreak strain, was very important for investigating the origin of the outbreak. Only three cases were diagnosed locally, and the investigation would have been very limited in the absence of information on the other cases. Sharing information on community outbreaks that also includes TALD cases allows for active case finding with helpful information on source identification. In this outbreak, the first ELDSNet notification was received on October 4, 2017, when twenty one of the twenty seven cases had already been infected (and had presented with symptoms). Although this frequently happens in outbreaks, it is necessary to emphasize the need to declare legionellosis cases as quickly as possible in order to be able to respond rapidly. Once again, the ELDSNet model, which collects data from all member countries of the network, makes it possible to quickly identify clusters of cases and promote rapid investigations and responses from local health authorities.

In this outbreak, the environmental investigations were key to identify its origin. In the absence of epidemiological data (most reported cases were tourists that had already returned to their country of origin), extensive sampling of possible sources allowed the source to be identified.

This outbreak investigation had several limitations. First, most of the cases were tourists from other countries, so it was not possible to obtain detailed questionnaires about their movements prior to becoming ill as having this information could have facilitated a clearer understanding about the distance travelled by contaminated aerosols. In the absence of this information, it therefore cannot be ruled out that the cases had been close to or even visited Hotel A. Secondly, it was not possible to investigate contaminated hot tubs usage by guests in the Hotel A penthouses. It is probable that some were used given that the occupancy of the penthouse on the sampling dates was 50% and that the proportion of hot tubs found positive for *Legionella* was very high. The fact that only one client of hotel A was affected is surprising but the hot tubs with the highest *Legionella* load were in the unoccupied rooms and their location, on the upper parts of the building, may have facilitated the spread of bioaerosols away from Hotel A. All infections occurred prior to hot tubs sampling, and it cannot be ruled out that the hot tubs in the occupied rooms were in a proper hygienic state. Indeed, it is possible that contamination levels fluctuate significantly over short periods of time (Wéry et al., 2008; Napoli et al., 2009), and even more in low volume systems with manual chemical dosing, thus making it difficult to draw conclusions on the water quality in the days when the infections occurred. In retrospect, we acknowledge that it would have been of interest to study the quality of the hot tub water in more depth, for example, analyzing the presence of *Escherichia coli*, *Pseudomonas aeruginosa*, amoeba and other physicochemical parameters, such as turbidity, which could have shed more light on the hygienic state of the hot tubs in question and perhaps on other aspects such as the pathogenicity or infectivity of the *Legionella* isolates.

No meteorological data was obtained at the time of the events and the data on the weather had to be obtained retrospectively. There was no weather data specifically from the affected area prior to the outbreak, although we believe that the data we have for the meteorological station of Son Ferriol (22 Km from Palmanova) and the Port of Palma de Mallorca (9 km from Palmanova) are quite representative of what the local conditions would have been in Palmanova. Indeed, the partial temperature and wind data that was obtained from the meteorological station located in the nautical club of the affected area was consistent with the above.

Our research shows that private outdoor hot tubs can disseminate aerosols distant from the premises in which they are located and raises the need to register these systems, especially when they are located in highly populated areas. It is also important that manufacturers and health authorities provide adequate instructions to operators and users for their safe use. In conclusion, our investigation shows that hot tubs for private use located outdoors should be considered as a potential source when investigating community outbreaks of LD of unclear origin and underlines the importance of adequate maintenance, water treatment and controls of hot tubs.

## Data availability statement

The raw data supporting the conclusions of this article will be made available by the authors, without undue reservation.

## Author contributions

SC, MG, and RC: conceptualization. SC, RC, MG, BS, and AN: data curation. SC, VD, RC, AN, MG, MB, JB, PC, BA, and BS: investigation and writing—review and editing. SC, VD, RC, MG, and AN: data analysis. SC: writing—original draft. All authors contributed to the article and approved the submitted version.

## Conflict of interest

The authors declare that the research was conducted in the absence of any commercial or financial relationships that could be construed as a potential conflict of interest.

## Publisher’s note

All claims expressed in this article are solely those of the authors and do not necessarily represent those of their affiliated organizations, or those of the publisher, the editors and the reviewers. Any product that may be evaluated in this article, or claim that may be made by its manufacturer, is not guaranteed or endorsed by the publisher.

## Footnotes

^1^ https://eportal.mapa.gob.es/websiar/SeleccionParametrosMap.aspx?dst=1

^2^https://windy.app/forecast2/spot/2648671/Club+N%C3%A1utico+Palmanova/statistics and https://es.windfinder.com/windstatistics/palma_de_mallorca_puerto

^3^ https://bioinformatics.phe.org.uk/legionella/legionella_sbt/php/sbt_homepage.php

^4^ http://legionella-sbt@ukhsa.gov.uk
